# A Deep-Learned Monolithic Nanoparticle Asymmetric Thermal Flow Sensor for Flow Vector Estimation

**DOI:** 10.1021/acsnano.5c07646

**Published:** 2025-08-12

**Authors:** Huijae Park, Sangjin Yoon, Junhyuk Bang, Jiyong Ahn, Gyuho Choi, Dohyung Kim, JinKi Min, Jaeho Shin, Seung Hwan Ko

**Affiliations:** † Wearable Soft Electronics Lab, Department of Mechanical Engineering, 26725 Seoul National University, 1 Gwanak-ro, Gwanak-gu, Seoul 08826, Korea; ‡ Molecular Recognition Research Center, Korea Institute of Science and Technology (KIST), Seoul 02792, South Korea; § Institute of Advanced Machinery and Design (SNU-IAMD), 26725 Seoul National University, Gwanak-ro, Gwanak-gu, Seoul 08826, Korea; ∥ Institute of Engineering Research, 26725 Seoul National University, 1 Gwanak-ro, Gwanak-gu, Seoul 08826, Republic of Korea; ⊥ Interdisciplinary Program in Bioengineering, 26725 Seoul National University, Gwanak-ro, Gwanak-gu, Seoul 08826, Korea

**Keywords:** laser processing, reduction sintering, asymmetric thermal flow sensor, heater, deep learning

## Abstract

Flow sensing is essential in various fields, including industrial, environmental, and biomedical applications, where accurate measurement of fluid dynamics is crucial. Traditional flow sensors are often bulky and complex, which can distort the flow and complicate installation when placed directly in the flow path. To address these issues, we developed a deep-learned monolithic asymmetric thermal flow sensor. The sensor is fabricated via laser-induced selective sintering and reduction of nickel oxide nanoparticles, seamlessly integrating a microheater and temperature sensors into a thin-film device. This thin-film design minimizes flow disturbance and improves measurement accuracy. Unlike conventional calorimetric flow sensors that require complex multiarray electrode configurations, our system features a temperature sensor designed in an asymmetric spiral shape around the heater. This optimized hardware configuration not only simplifies the structural design but also supports deep learning algorithms for accurate flow estimation. By integrating this asymmetric design with reinforcement learning algorithms, the sensor efficiently bridges hardware and software, enabling precise flow vector estimation based on changes in sensor resistance. Furthermore, equipped with an embedded wireless communication system for real-time data monitoring, the sensor ensures reliable flow assessment, making it a versatile solution for diverse flow estimation applications.

## Graphical abstract

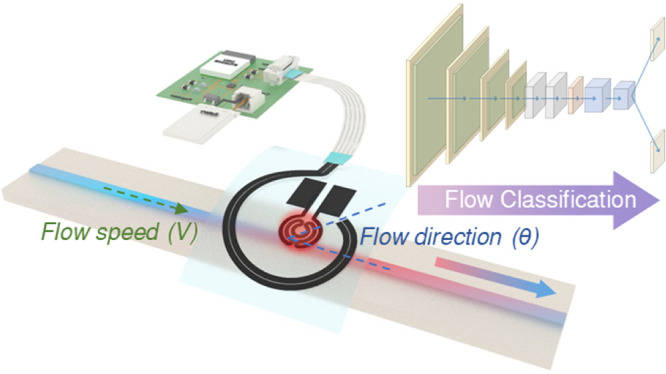

## Introduction

Thermal energy transfer is a fundamental physical phenomenon that plays a crucial role in various scientific and engineering applications. It involves the movement of heat from one location to another, driven by temperature differences, and occurs through three primary mechanisms: conduction, convection, and radiation. Accurate analysis and control of thermal energy transfer are essential for optimizing energy efficiency, maintaining thermal stability, and improving the performance of numerous systems. The broad significance of heat transfer has inspired the development of advanced sensing technologies that leverage thermal principles for precise measurements and monitoring.

Flow sensing is a critical technology for detecting and quantifying fluid motion in diverse environments, ranging from industrial processes to biomedical applications. Various flow sensing methods have been developed based on different physical principles, including mechanical,^1–3^ optical,^4–6^ acoustic-ultrasonic,^7,8^ electromagnetic,^9–12^ and thermal mechanisms.^13–17^ Among these, thermal flow sensors are particularly noteworthy due to their high sensitivity, simple mechanism, and capability for noncontact measurements.

Thermal flow sensing technologies have continued to evolve with the integration of advanced materials and fabrication techniques. Calorimetric flow sensors, for example, utilize the principles of heat transfer to accurately measure fluid flow speed and directions. These sensors are commonly applied in diverse fields such as environmental monitoring,^18–21^ biosignal detection,^22–26^ aerospace,^27,28^ and precision manufacturing processes.^29–31^ By capturing temperature changes in the fluid, they provide nonintrusive and highly precise flow measurements, which is essential in applications where direct flow rate measurement is challenging. However, conventional calorimetric flow sensors often rely on complex multiarray electrode configurations.^20,21^ This intricate structure poses challenges in data acquisition and analysis,^28^ and has also been identified as a limiting factor for miniaturization in systems requiring compact integration.^32^ Consequently, such bulky and complex sensors can be difficult to deploy in scenarios where a compact and simplified design is required.

To overcome these limitations, we developed a novel monolithic asymmetric thermal flow sensor that addresses the challenges of conventional flow sensors. Utilizing a laser-induced selective reduction process to sinter and pattern NiO nanoparticles, we achieved monolithic integration of both the heater and the temperature sensor on a single substrate. Notably, the laser-based selective reduction process, typically studied for phase control of various materials,^33–37^ is innovatively applied here to fabricate an integrated thin-film flow sensor. The sensor’s thin-film design minimizes flow distortion and enables device miniaturization, ensuring accurate and reliable measurements without significantly interfering with the flow. This integrated approach provides high sensitivity in a compact form factor, allowing for precise flow measurements in situations where traditional sensors would be impractical.

A key innovation of our sensor lies in its optimized hardware configuration. Unlike conventional calorimetric flow sensors that require multiple sensing elements or electrode arrays, our design features an asymmetric spiral temperature sensor pattern around a central heater. This structural simplicity facilitates a smaller device footprint while still capturing rich information about the flow. We further leverage deep learning algorithms to interpret the sensor data. The asymmetric hardware design combined with advanced neural network models enables accurate determination of flow direction from a single sensor’s resistance change. The ability to derive dynamic flow vector information from a monolithic asymmetric thermal flow sensor highlights the novelty of our approach. To the best of our knowledge, this study is the first to apply a laser-induced selective reduction process to create a monolithic asymmetric thermal flow sensor, representing a significant advancement in flow sensing technology.

Figure 1a provides an overview of the monolithic asymmetric thermal flow sensor module. The liquid flows through a channel above which the module, featuring a circular heater and temperature sensors, is positioned. As the liquid passes through the channel, the heater experiences convective heat loss proportional to the flow speed. At the same time, heat is carried downstream by the fluid and is detected by different parts of the asymmetric spiral temperature sensor, providing information about the flow direction. This information is transmitted wirelessly via data transmission module, with the heater’s power controlled through pulse-width modulation (PWM). The resistance change data is acquired through an Analog-to-Digital Converter (ADC), and based on this data, a deep learning model classifies the flow into its speed and direction components. Figure 1b illustrates the heat transfer mechanism in the sensor. The heater is a circular pattern of NiO that has been fully laser-reduced to metallic nickel. The temperature sensing element is a Negative Temperature Coefficient (NTC) thermistor made of NiO that remains in its semiconducting oxide form. This spiral-shaped NiO sensor surrounds the heater and is connected by monolithic nickel electrodes on either side. The heater and the sensor are patterned on opposite sides of the PET substrate to minimize mutual interference. As the heated liquid flows along the channel, heat distribution occurs, causing temperature changes, in the asymmetric spiral sensor surrounding the heater. These changes are then used to evaluate the dynamic flow characteristics of the liquid. Figure 1c shows the architecture of the deep learning model that processes the sensor data. The model takes the time-series temperature (*T*) data as input and passes it through several computational layers. Convolutional layers and MaxPooling layers extract spatial features from the temperature signal, residual blocks help deepen the network and capture complex patterns, and a long short-term memory (LSTM) layer captures temporal dynamics in the changing sensor readings. Finally, fully connected layers output the predicted flow direction (θ) and speed (*v*). By integrating wireless data communication with this deep learning framework, our sensor system enables real-time monitoring and classification of flow conditions. This seamless combination of innovative hardware design and advanced data processing enhances measurement accuracy while simplifying the overall system architecture, making it a versatile and promising solution for a wide range of flow sensing applications.

**Figure 1:**
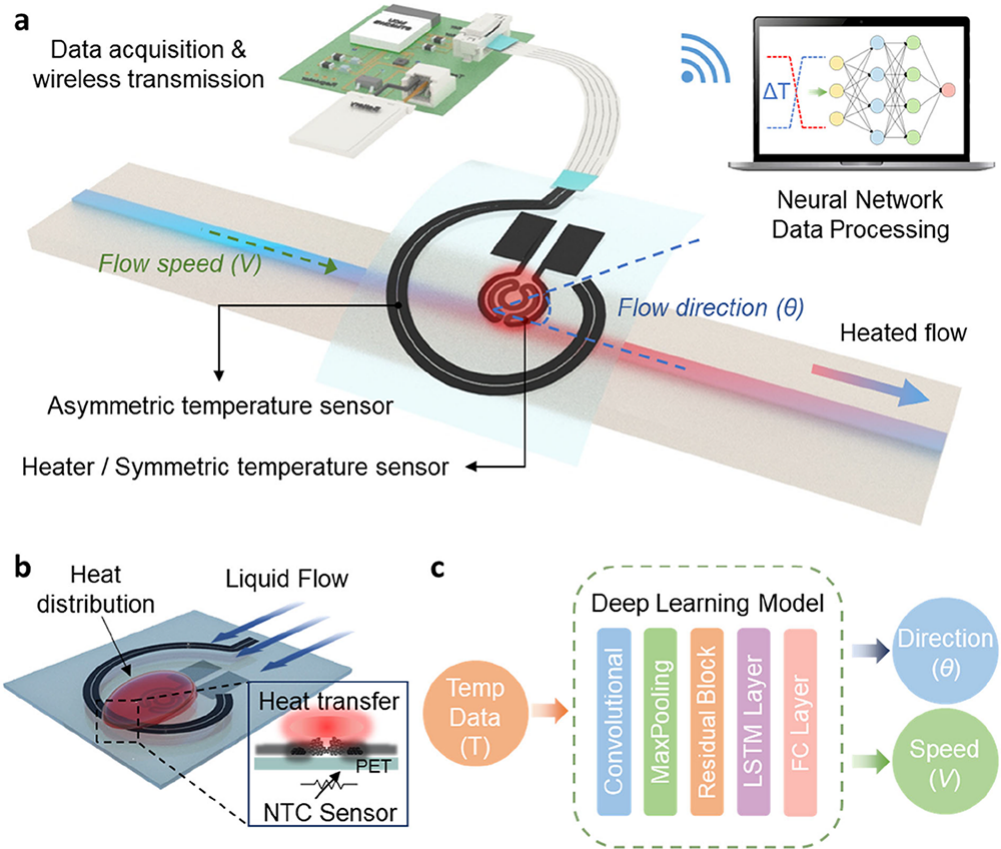
Schematics of the sensor’s operation mechanism and data processing workflow. (a) Overview of the monolithic asymmetric thermal flow sensor module in a flow channel. The sensor measures flow speed by detecting the heat loss from the circular Ni heater, and it determines flow direction by using the asymmetrically patterned NiO temperature sensor around the heater. Data acquisition is performed via an ADC on an embedded MCU, and the data is transmitted wirelessly. The heater power is controlled through PWM by the MCU. The collected resistance data is processed by a neural network to classify the flow’s speed and direction. (b) Schematic of heat distribution changes induced by liquid flow. As the liquid heated by the heater flows along the flow channel, heat is transferred to the asymmetrically patterned NTC temperature sensors around it, resulting in a change in resistance. (c) Architecture of the deep learning model for flow prediction. The model processes temperature data (*T*) using a combination of Convolutional, MaxPooling, Residual Block, LSTM, and Fully Connected layers. The final output predicts flow direction (θ) and speed (*V*) using deep learning algorithms.

## Results and Discussion

As shown in Figure 2a, PVP-coated NiO nanoparticles are uniformly deposited on a PET substrate, forming a thin and even layer. When a laser is scanned over this film under optimized conditions, the directly irradiated regions are simultaneously reduced and sintered into metallic nickel, while the surrounding areas, which are not directly exposed to the laser focus, remain as NiO and are thermally sintered by the laser’s heat. The optical setup of the laser system was configured as shown in Figure S1. By using a visible-wavelength laser, the reduction of nickel oxide nanoparticles to metallic nickel was induced. Additionally, the CAD files were iteratively modified, allowing us to rapidly optimize the design of the heater and sensor. Laser patterning was performed at the focal plane of the laser, with a slight defocus of about 0.5 mm to prevent ablation of the material. In Movie S1, the top surface of the substrate is patterned with a circular heater, while the bottom surface features asymmetric and symmetric temperature sensors. Figure 2b illustrates the reduction mechanism of PVP-coated nickel oxide nanoparticles. The nanoparticles are stabilized by PVP, which prevents aggregation and ensures uniform distribution. Upon laser irradiation, the photothermal effect generates heat, weakening the bonds between metal ions and oxygen, leading to oxygen release. Concurrently, the PVP on the nanoparticle surface thermally decomposes and can act as a reducing agent, absorbing the released oxygen and thus facilitating the reduction of NiO to Ni. As a result, in the laser-irradiated regions the NiO particles are chemically reduced to metallic Ni and simultaneously sintered together into a conductive path.^38^ On the other hand, if the laser’s energy in a region is insufficient to induce the reduction reaction, such as in areas that receive only indirect or scattered light, no Ni metal is formed. In these regions, the NiO nanoparticles still experience heat, which is enough for the PVP to melt and the NiO particles to sinter together, but they remain in the NiO phase. These unreduced NiO areas function as the temperature-sensing elements. Figure S2 illustrates the overall fabrication process of the monolithic asymmetric thermal flow sensor. NiO/PVP ink is coated onto a PET substrate, forming a circular heater on the top side, while temperature sensors are fabricated on the bottom side through a selective reduction process. In detail, Figure S3 provides a detailed illustration of how NiO ink is coated and patterned on the opposite side of the PET substrate to fabricate the thermal flow sensor. To ensure accurate alignment between the heater and the sensor, rectangular reference markers were patterned around both components. During the bottom-side patterning process, the substrate was secured at its corners on the stage, and the markers on the heater side were used as visual guides to precisely align the features on both sides. In Figure 2c, the bandgap of the material was measured to confirm its semiconductor properties and NTC functionality. The bandgap was determined to be 3.35 eV, verifying its semiconductor characteristic of decreasing resistance with increasing temperature.^39–41^ Figure 2d presents the Raman shift measurements of the PVP-coated nickel oxide nanoparticles and the sintered nickel oxide temperature sensor, taken after the PVP coating has melted away. When observing the Raman shift peak of the sensing channel, the carbon D and G peaks become more pronounced compared to the pristine spectrum without laser treatment. This enhancement is attributed to the thermal decomposition and carbonization of PVP under laser exposure, which produces reducing agents and carbonaceous structures, resulting in the appearance of characteristic carbon D and G peaks in the Raman spectrum of the laser-treated region.^23^ Additionally, a ψ peak shift from 500 to 550 cm^–1^ can be observed, which can be attributed to the attenuation of defect-activated modes and the enhancement of stoichiometric longitudinal modes, resulting from a reduction in crystalline defects under reductive thermal annealing conditions induced by the laser process.^42,43^ Figure 2e compares the X-ray diffraction (XRD) measurements of the reduced nickel with those of the unreduced nickel oxide, highlighting the structural differences. The differences in XRD peaks between nickel oxide and metallic nickel originate from their distinct crystal structures and crystallinity. Nickel oxide, with a face-centered cubic (FCC) rock-salt structure, exhibits peaks at approximately 37.2° (111), 43.3° (200), and 62.9° (220). These peaks are relatively broad due to nanoscale crystallite size, structural defects, and internal lattice strain.^44^ In contrast, reduced nickel also forms an FCC structure, but presents sharp and intense peaks at 44.5° (111), 51.8° (200), and 76.4° (220), which reflect high crystallinity and well-ordered atomic arrangements.^45^ Notably, the (111) peak of nickel at 44.4° lies close to the (200) peak of NiO at 43.3°, potentially complicating phase identification in mixed-phase systems. In Figure 2f, atomic force microscopy (AFM) measurements of the nickel and nickel oxide demonstrate a seamless connection between the temperature sensor and the electrodes. In the AFM image, the brighter surrounding regions are interpreted as metallic nickel, while the darker areas correspond to nickel oxide. The degree of sintering was confirmed through AFM for the areas that were not laser-irradiated, the parts indirectly annealed where only PVP was melted without phase change, and the parts reduced to Ni through the selective reduction process (Figure S4). The root-mean-square roughness (Rq) of the pristine ink was measured as 125.412 nm, the indirectly sintered area was 166.254 nm, and the directly irradiated and reduced area was 568 nm. This shows that the degree of sintering of the nanoparticles increases with the extent of laser irradiation, leading to an increase in nanoparticle size and a change in roughness. Figure 2g shows scanning electron microscope (SEM) images of the sensor module, where the central region functions as the temperature sensor, flanked by nickel electrodes on either side. The temperature sensor region was patterned with a 50 μm hatch spacing, while the electrode regions were reduced using a 20 μm hatch spacing under laser irradiation. Finally, Figure 2h displays energy-dispersive X-ray spectroscopy (EDS) images, indicating that the nickel oxide temperature sensor area has a concentrated oxygen distribution, while the reduced nickel regions are devoid of oxygen, confirming the success of the reduction process. Notably, the presence of oxygen even in the reduced nickel regions can be attributed to the PET substrate. Since the chemical formula of PET is (C_10_H_8_O_4_)_*n*_, oxygen is inherently present throughout the substrate. Consequently, a certain amount of oxygen is still detected in the reduced regions due to this PET background. The SEM and EDS images further confirm that the NiO and Ni sections are patterned continuously and monolithically. A parameter study was conducted to assess the degree of reduction based on laser power. Among the tested conditions, lower speeds combined with higher power led to burning of the PET substrate, while higher speeds resulted in weak adhesion and pattern removal during cleaning. The selected condition also exhibited superior mechanical durability compared to other settings (Figure S5). As shown in Figure S6, the lowest sheet resistance was observed at a laser power of 100 mW, with the laser speed fixed at 5 mm/s during the experiment. To prevent nanoparticle ablation, the substrate was slightly defocused by about 0.5 mm, so the laser was not directly focused on the surface. Under these conditions, the most effective reduction was achieved.

**Figure 2:**
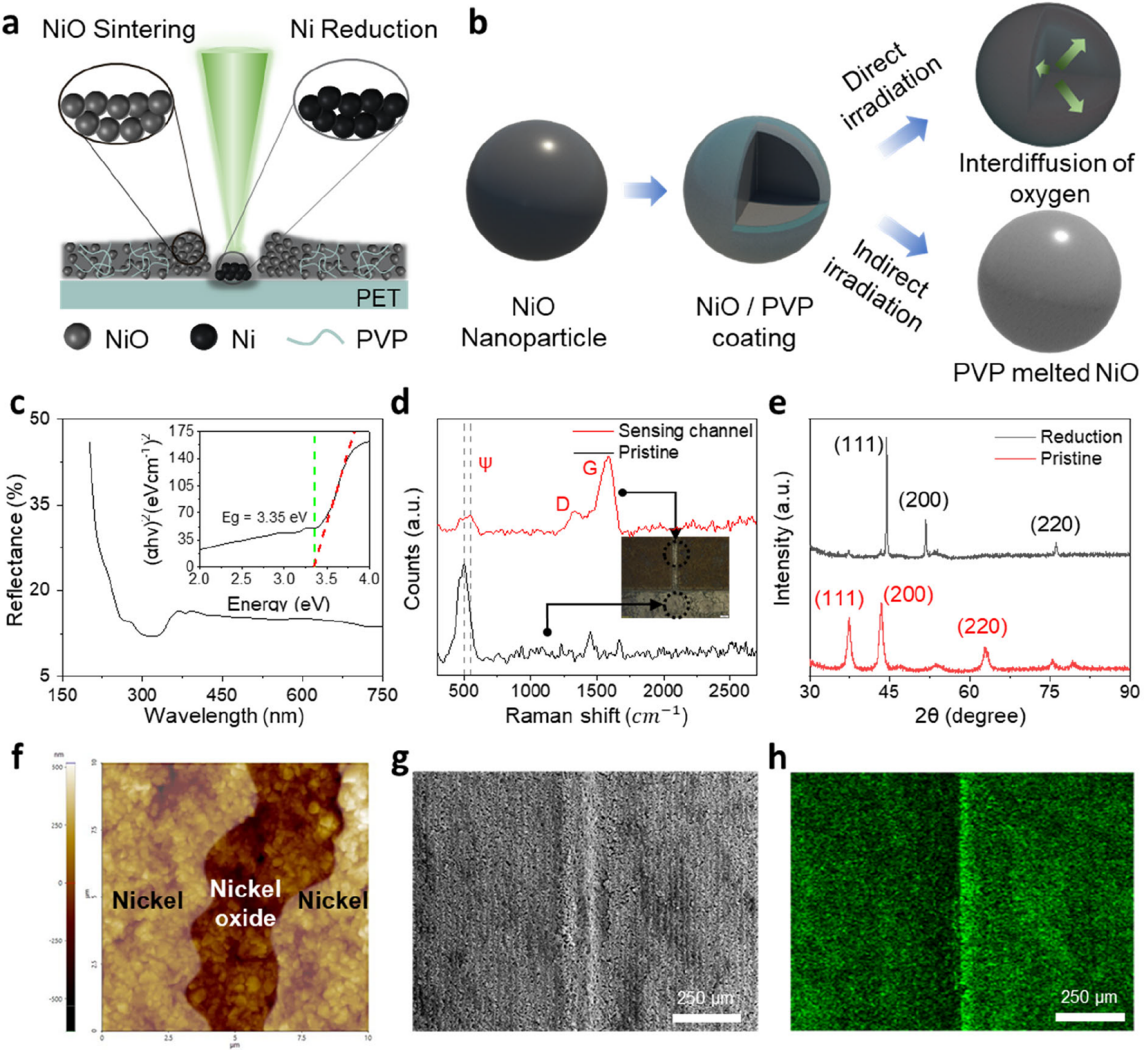
Reduction mechanism and characterization of nickel oxide nanoparticles through laser processing. (a) Schematic of laser-induced reduction and sintering of nickel oxide nanoparticles. The laser-irradiated region is reduced to nickel, while the surrounding area undergoes only sintering without reduction. (b) Reduction mechanism of PVP-coated nickel oxide nanoparticles. Laser irradiation generates heat via the photothermal effect, breaking bonds between metal ions and oxygen, resulting in oxygen release and metal ion reduction. PVP stabilizes the nanoparticles and facilitates oxygen removal. If the generated heat is insufficient, only sintering occurs as PVP melts without reduction. (c) Optical band gap of NiO determined as 3.35 eV using Tauc plot analysis. (d) Raman spectra of the NTC NiO temperature sensor and the pristine solution ink. (e) Comparison of XRD pattern between Ni and NiO. (f) AFM analysis of nickel and nickel oxide after sintering. (g) SEM image of the laser reduced Ni electrode and sintered NiO temperature sensor. (h) EDS mapping of the Ni electrode and NiO temperature sensor. The oxygen distribution is concentrated in the NiO temperature sensor area in the middle.

Figure 3a provides a simplified schematic of the interaction between the heater and the flowing liquid in our experiment. When the heater is powered via PWM control, it generates heat, raising its temperature. In the presence of liquid flow beneath the heater, the heater continuously loses some of its heat to the moving fluid. The heated fluid, in turn, carries that heat downstream along the channel. Figure 3b presents a graph showing the heater’s performance, with temperature measured using an IR camera while incrementally increasing the voltage by 0.5 V. The temperature rose linearly with increasing voltage, reaching approximately 130 °C at 5 V. Inset images illustrate the IR measurements at each voltage level. Figure 3c highlights the heater’s stability under extended operation. Voltage was applied in 1 V increments for 10 min intervals, during which the heater maintained a stable temperature, demonstrating consistent performance. Stable operation of the heater is important because it ensures that any changes in sensor readings are due to external influences rather than fluctuations in the heater output. Figure 3d displays the results of a cyclic durability test, where a voltage of 0.1 W was repeatedly applied and removed. The voltage was applied for 40 s and then turned off for 40 s, with this cycle repeated approximately 100 times. During each on–off cycle, the heater consistently heated and cooled to specific temperatures, confirming its reliability and durability under prolonged and repetitive use. Figure 3e shows the heat distribution results from the IR camera, reflecting the flow of the liquid beneath the heater. The measurements were taken at flow rates increasing in 0.1 mL/min increments. As the flow rate increased, significant heat loss occurred in the central region of the heater, while the heated liquid was observed moving along the flow channel, forming a distinct heat distribution pattern. As shown in Movie S2, as the flow speed increases, the heat generated by the heater quickly forms a thermal distribution along the flow direction, while heat loss occurs directly above the heater. In Figure 3f, a simulation was conducted using COMSOL Multiphysics to predict the heat loss of the heater and the temperature distribution of the heated liquid. The heat transfer was modeled by solving the steady-state heat transfer equation in fluids and solids, omitting the time-dependent term.

$$\rho c_{p}(\partial T/\partial t+u\cdot \nabla T)=\nabla \cdot (k\nabla T)+Q$$ 1

where ρ is the density, *c*_p_ is the specific heat capacity, *T* is the temperature, *u* is the velocity field, *k* is the thermal conductivity, and *Q* is the volumetric heat source. The heater power was adjusted so that the simulated temperature matched the experimental IR camera measurements, ensuring consistency between the model and actual device behavior. The inlet water temperature was set to 25 °C, and all boundaries were assumed adiabatic. The mesh size ranged from 5.43 to 310.14 μm, with finer meshing applied near the heater and fluid–solid interfaces to resolve steep temperature gradients. The resulting temperature profile showed good agreement with experimental observations, validating the modeling approach. Based on this phenomenon, a temperature sensor capable of measuring heat loss at the heater’s central region was patterned directly beneath the heater, enabling the measurement of fluid flow speed. Additionally, to detect changes in heat distribution along the channel due to fluid flow, an asymmetric spiral-shaped temperature sensor, whose distance from the heater varies with angle, was patterned around the heater, thus providing a means to classify the direction of fluid flow.

**Figure 3:**
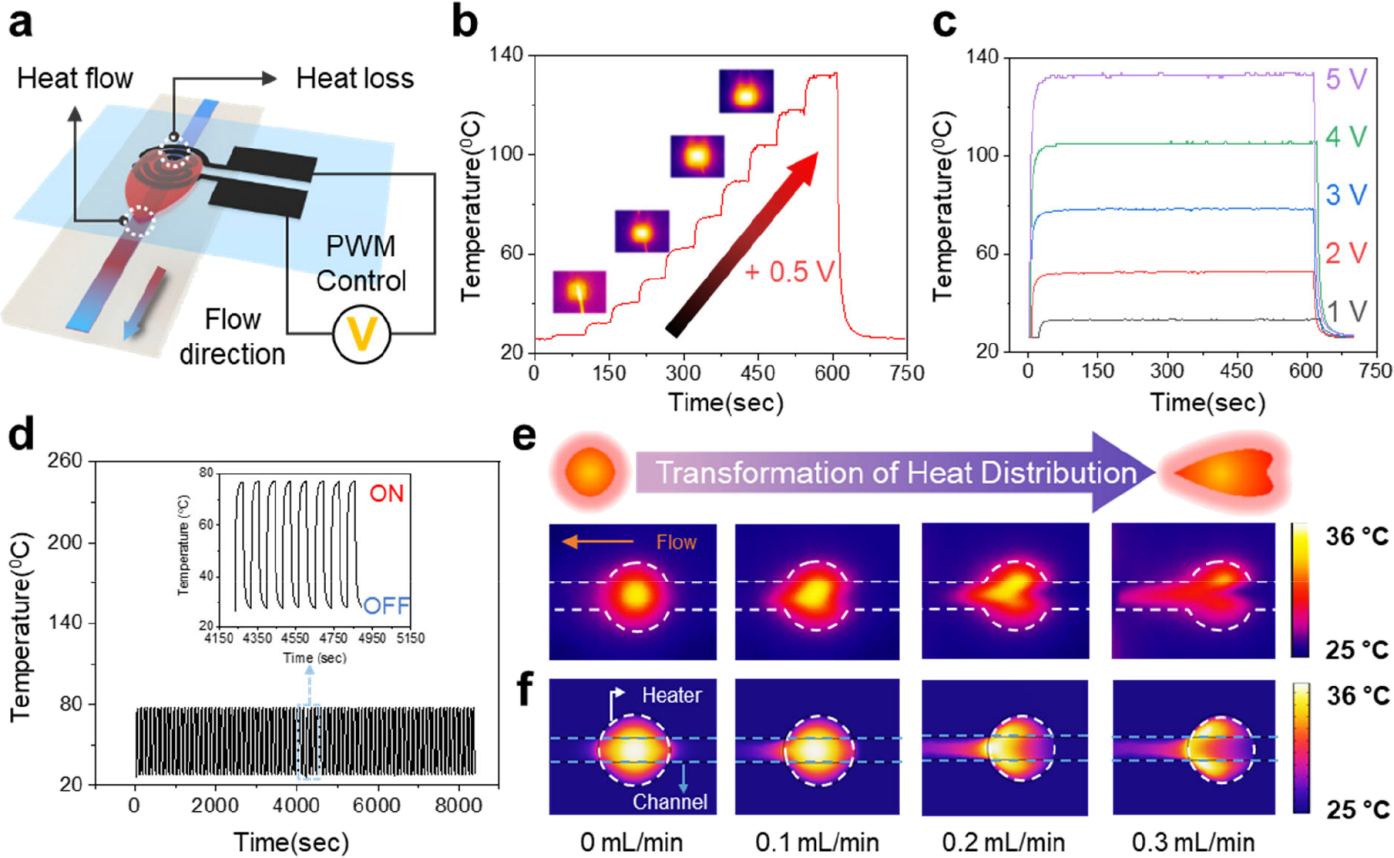
Schematics of heater performance and heat distribution. (a) Schematic diagram illustrating heat loss from the heater. and heat distribution along the flow channel. (b) Heater temperature observed as voltage increases in 0.5 V increments. The inset image shows the heater’s temperature as measured by an IR camera. (c) Real-time temperature variation of the heater when voltage is applied for an extended period in 1 V increments. (d) Temperature changes of the heater when a constant power is repeatedly applied. (e) Infrared thermal images showing heat distribution during fluid flow at intervals of 0.1 mL/min. (f) Simulation results corresponding to the heat distribution experiment.

As depicted in Figure 4a, the schematic outlines the arrangement of the heater and temperature sensors. The top layer features a circular heater patterned on a PET substrate. Directly beneath the heater, on the same substrate, lies a central temperature sensor designed to measure heat loss from the heater to determine the fluid flow speed. Additionally, an asymmetrically patterned temperature sensor is positioned around the heater’s periphery, enabling detection of changes in the heater’s heat distribution caused by fluid flow, thereby identifying the direction of the flow. The size optimization of the asymmetric temperature sensor was carried out based on the results shown in Figure S7. It was observed that the resistance changes gradually decreased as the distance from the heater increased. Accordingly, the spiral temperature sensor was patterned to span from a minimum distance of 1 mm to a maximum distance of 9 mm from the heater. Detailed geometrical information on the heater and temperature sensor is provided in Figure S8. To construct the integrated device, a 0.4 mm-thick PDMS layer was first spin-coated onto a glass substrate and patterned to form a 1 mm-wide flow channel. A thin polyurethane film (Tegaderm, 3M) was then attached onto the PDMS layer to serve as an insulating barrier (Figure S9). In addition to serving as a physical insulation layer, the Tegaderm film also acted as an environmental barrier that mitigates interference from ambient humidity chemical species such as CO_2_, which could otherwise cause resistance drift in NiO sensors.^23^ Finally, the PET film with the patterned heater and temperature sensors was carefully aligned and gently pressed onto the polyurethane surface without permanent bonding. This stacked configuration allowed efficient thermal interaction with the flowing fluid while protecting the sensor from direct contact. Figure 4b illustrates the mechanism of the temperature sensor’s data variation. It shows a cross-sectional view of the liquid flowing along the channel and a top view of the asymmetric thermal flow sensor. The central temperature sensor measures resistance changes caused by heat loss from the heater, enabling the flow rate measurement. The asymmetrically patterned sensor, with varying distances from the heater, measures heat distribution, allowing the determination of the liquid’s flow direction. Figure 4c shows the performance of the temperature sensor. It also analyzes the relationship between resistance and temperature variation based on the laser hatch spacing. When the spacing is less than 50 μm, the electrodes connect, preventing the sensor from functioning as an NTC temperature sensor. Instead, it exhibits the characteristic behavior of metal electrodes, where resistance increases with temperature. Conversely, when the spacing exceeds 50 μm, the electrodes remain unconnected, and the sensor operates as an NTC sensor, where resistance decreases as temperature increases. Figure S10 shows that as the hatch spacing increases to 70 μm, the sensitivity decreases compared to that at 50 μm. Although the sensor with a 40 μm hatch spacing exhibits metallic behavior, the resistance change is unstable. These results indicate that a hatch spacing of 50 μm provides the most optimal performance. Figure 4d presents a graph showing the sensor’s high sensitivity to temperature changes. The *B*-value of an NTC sensor represents the material’s sensitivity to temperature changes and is defined by the relationship between resistance and temperature. It can be calculated using the following formula.^46,47^

$$B=\frac{\mathrm{In}\left(\frac{R_{1}}{R_{2}}\right)}{\frac{1}{T_{1}}-\frac{1}{T_{2}}}$$ 2

where *R*_1_ and *R*_2_ are the resistances at temperatures *T*_1_ and *T*_2_ (K). A higher *B*-value indicates a greater change in resistance per degree of temperature change, making the sensor more responsive to variations in temperature. The *B*-value is 3944 K in the temperature range of 25–70 °C, while it increases significantly to 8273 K in the range of 25–33 °C, indicating a highly sensitive response. This suggests that the temperature sensor can detect temperature changes with high sensitivity within this range, particularly when water is heated and flows at room temperature due to heat distribution. Figure 4e evaluates the sensor’s durability by measuring resistance under repeated temperature variations. The experiment involved heating to 70 °C and then returning to room temperature, with this cycle repeated approximately 150 times. The results confirm consistent resistance changes, demonstrating the sensor’s reliability. The mechanical reliability of the sensor was further evaluated by subjecting it to 300 bending cycles at a radius of 5.18 mm. As shown in Figure S11, the resistance exhibited periodic fluctuations without noticeable drift, indicating the sensor’s durability under flexible conditions.

**Figure 4:**
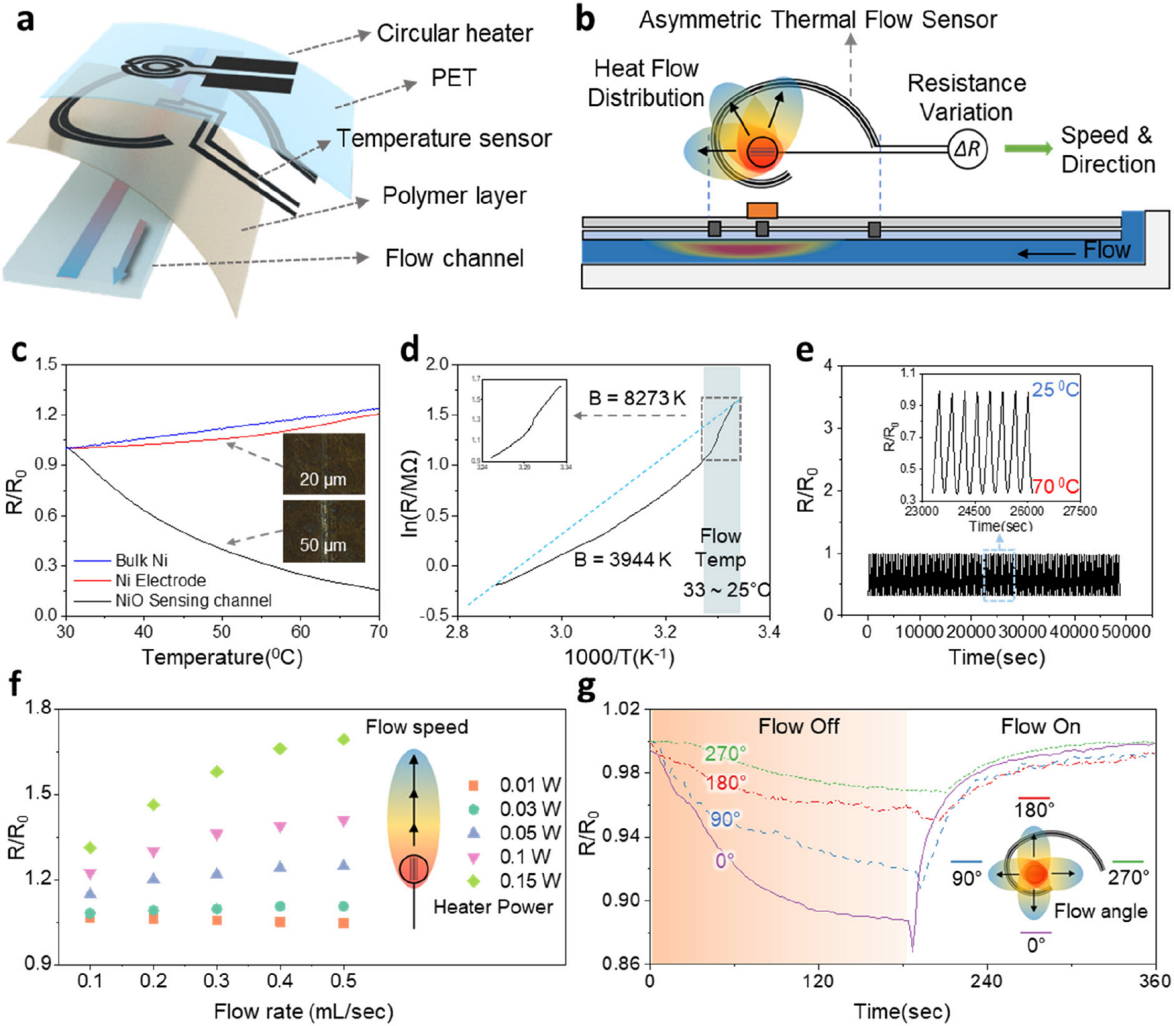
A schematic illustrating the composition, working principle, and performance of the sensor module. (a) Layered structure of the flow sensor. The PET substrate has a circular heater on the top, a temperature sensor on the bottom, and a thin polymer base. (b) Cross-sectional and top views of the sensor, showing how the central temperature sensor detects resistance changes due to heat loss, enabling flow rate measurement. (c) Resistance changes of PTC and NTC with temperature. A gap of less than 50 μm exhibits PTC characteristics due to electrical shorting, while a gap greater than 50 μm shows NTC characteristics. (d) Fitting for the material constant of the thermistor (B-value) revealed an exceptionally high B-value of 3944 K across the entire measurement range (25–70 °C) and 8273 K within the room temperature range (25–33 °C). (e) Sensor durability assessment through resistance measurements under repeated temperature cycles. The sensor was heated to 70 °C and cooled to room temperature for approximately 150 cycles. (f) Resistance variations as a function of heater power and flow speed. At low heater power, insufficient heat transfer to the flowing water results in minimal resistance variation. At higher heater power, heat loss with flow rate changes, leading to noticeable resistance shifts. (g) Resistance decreases when the flow stops (Flow Off) due to heat accumulation. Upon flow activation (Flow On), a sharp resistance peak appears, followed by stabilization. Smaller angles show greater resistance changes due to closer proximity to the heater, while larger angles exhibit reduced variations.

The heat transferred from the heater to the water equals the amount of heat required to induce the observed temperature change in the flowing water. This relationship can be expressed by the following equation.

$$Q_{h}=U\cdot A\cdot (T_{h}-T_{\mathrm{avg}})=ṁ\cdot C_{p}\cdot (T_{\mathrm{out}}-T_{\mathrm{in}})$$ 3

*Q*_h_ represents the heat transferred from the heater to the water (J/s), *U* is the overall heat transfer coefficient (1698.64 W/m^2^·K), *A* is the heat transfer area (4 × 10^–7^ m^2^), and *T*_h_ is the temperature of heater (80 °C). *ṁ* denotes the mass flow rate of the water (kg/s), and *C*_p_ is the specific heat of water (4184 J/(kg·°C)). *T*_in_ is the inlet water temperature (25 °C), and *T*_out_ is the outlet water temperature.

$$T_{\mathrm{avg}}=\frac{T_{\mathrm{in}}+T_{\mathrm{out}}}{2},\;m˙=\rho \cdot V\cdot A$$ 4

*T*_avg_ is the average temperature of the water, ρ represents the water density, which is 1000 kg/m^3^ and *V* denotes the water velocity.

$$U=\frac{1}{\frac{1}{h_{\mathrm{water}}}+\frac{T_{\mathrm{pet}}}{K_{\mathrm{pet}}}},\;h_{\mathrm{water}}=\frac{Nu\cdot k}{D_{h}}=\frac{3.66\cdot k}{D_{h}}$$ 5

*T*_pet_ represents the thickness of PET (20 × 10^–6^ m), *K*_pet_ is the thermal conductivity of PET (0.15 W/(m·K)), *h*_water_ is the heat transfer coefficient of water (2196 W/(m^2^·K)), *k* is the thermal conductivity of water (0.6 W/(m·K)), and *D*_h_ is the channel width (1 mm). In this analysis, assuming laminar flow and a constant wall temperature condition, the Nusselt number was taken as 3.66.

Re=ρ·V·Dhμ,Pr=μ·Cpk,Nu=3.66 6

μ represents the dynamic viscosity of water. The Nusselt number (*Nu*) is generally expressed as a function of the Reynolds number (*Re*) and the Prandtl number (*Pr*). The internal flow behavior of water can be described using the above equation.

$$T_{\mathrm{out}}=\frac{2U\cdot A\cdot T_{h}+(2\rho \cdot Q\cdot C_{p}-U\cdot A)\cdot T_{\mathrm{in}}}{2\rho \cdot Q\cdot C_{p}+U\cdot A}$$ 7

By organizing the equation and analyzing the change in *T*_out_, it is observed that it varies linearly with the heater temperature (*T*_h_) and follows a power-law relationship with changes in flow rate (*V*). In our calculations, we assume that the thermal conductivity, heat capacity, and viscosity of water remain constant over the relevant temperature range. This assumption is justified by the relatively narrow temperature variation during operation. Although these material properties are known to be temperature-dependent, their variation within this range is modest and does not significantly affect the trend or validity of the derived relationships. Nonetheless, for applications involving broader temperature fluctuations or fluids with higher sensitivity to temperature, incorporating temperature-dependent property models may improve prediction accuracy. A comparison between the theoretical and experimental values is presented in Figure S12. When the flow rate was fixed and the heater power was varied, the resistance changed linearly. Conversely, when the heater power was fixed and the flow rate was varied, the resistance exhibited power-law behavior.

Figure 4f illustrates the trend of resistance changes in response to variations in heater power and flow speed. When the heater power is too low, the heat transfer to the flowing water is insufficient, resulting in minimal resistance changes with respect to flow speed. However, when the heater power is high enough to transfer sufficient heat to the flowing water, the amount of heat loss due to flow speed changes become significant. Figure 4g shows the trend of resistance changes in the asymmetric sensor based on the flow direction. When the flow is deactivated (Flow Off), the liquid gradually slows down and eventually stops, allowing the heater to warm the surrounding area and causing the resistance to decrease. As the angle decreases, the distance to the heater shortens, and heat transfer through the water in the channel beneath the asymmetric thermal flow sensor becomes more pronounced, resulting in a steeper resistance gradient. When the flow is activated (Flow On), a sudden peak in resistance change occurs at the initial activation point. This peak is caused by the movement of heated water along the channel, which induces heat transfer and temporarily disrupts the thermal equilibrium. As the angle increases and the distance from the heater becomes greater, the peak magnitude and overall resistance change decrease. This is because sensors located farther from the heater experience slower temperature changes, resulting in less pronounced resistance variations. Based on this trend, data was collected repeatedly, and deep learning was performed to classify the direction of the water at 45° intervals.

Figure 5a illustrates a flowchart of the overall flow sensor system, showcasing its integration of hardware and software components for precise data acquisition and analysis. The resistance of the temperature sensor is measured via an ADC connected to a microcontroller unit (MCU), while the heater is controlled through PWM. An additional battery powers the MCU, and a reference temperature sensor measures the initial liquid temperature. Data is collected and monitored on a laptop through Bluetooth wireless communication, enabling seamless transfer of information. The collected data is then used to train a sophisticated deep learning framework, enabling accurate classification of the flow vector. Figure 5b presents a photograph of the fabricated wireless communication module, where the embedded system, connected to a battery and an asymmetric thermal flow sensor, is positioned above the flow channel. The inset image showcases a laser-patterned asymmetric thermal flow sensor. The circuit design schematic of the embedded system and a photograph of the experimental setup are presented in Figure S13. Additionally, a video demonstrating real-time wireless data transmission from the sensor and monitoring via the GUI is provided (Movie S3). Figure 5c illustrates the convolutional neural network (CNN) layer schematic, which is a core component of a deep learning framework designed to classify angular and speed-related characteristics from resistance-based experimental data. The model combines CNNs, residual blocks, and LSTM layers to effectively capture both spatial and temporal patterns. Key statistical features, such as peak ratio, number of peaks, standard deviation, and skewness, are extracted and normalized for integration with sequence data. The CNN consists of four 1D convolutional layers with 64, 128, 256, and 512 filters, each followed by batch normalization and ReLU activation. Two residual blocks with 512 filters refine feature representations. The output is passed to a three-layer LSTM with 256 hidden units to model temporal dependencies. Additional features are concatenated with the final LSTM output and processed through two fully connected layers (1024 and 512 units) with dropout. The model has two output heads for classifying angle and speed using softmax activation. The model was trained using the AdamW optimizer (learning rate = 10^–5^, weight decay = 10^–5^) with a cosine annealing scheduler. A batch size of 256 was used over a maximum of 5000 epochs, with early stopping based on validation loss (patience = 20,000). Cross-entropy loss was used for both outputs, and data were split into 80% training and 20% validation sets. This framework demonstrated robust classification performance by combining hierarchical feature extraction and temporal modeling, while regularization techniques helped prevent overfitting. Figure 5d illustrates the trends in the data based on flow speed and direction, using a scatter plot to visualize resistance data after dimensionality reduction with t-Distributed Stochastic Neighbor Embedding (t-SNE). This method effectively maps high-dimensional data into a 3D space while preserving the relationships between samples. Each point in the plot represents a data sample, positioned according to the t-SNE-reduced dimensions and color-coded by angle class using a distinct colormap for clarity. The axes, labeled as “t-SNE Component 1”, “t-SNE Component 2”, and “t-SNE Component 3”, provide a spatial framework for analyzing the distribution and clustering of the data. This visualization highlights the separability and structure of the data, revealing patterns and relationships among the classes. Figure 5e further supports the relationship between heat distribution and flow dynamics by presenting the results of the deep learning analysis, which show a classification accuracy of 100% for flow speed and 96% for flow direction, thereby demonstrating the model’s effectiveness. The analysis was performed with a maximum of 5000 epochs, and it was observed that the accuracy converged to its maximum value after approximately 5000 iterations. More specifically, the loss and accuracy values over epochs were presented for the training process of flow speed and direction. Figure S14 illustrates the loss values for both flow direction and speed during training, plotted against the number of epochs. It was observed that the loss for direction decreased more gradually compared to speed. Additionally, Figure S15 presents the accuracy values for flow direction and speed over epochs, showing that the training reached a stable accuracy after approximately 5000 epochs. As a result, the validation data were evaluated and visualized using a confusion matrix, which enabled a quantitative assessment of the model’s accuracy. With further training on larger datasets and advancements in deep learning models, it is expected that the system will be able to infer continuous fluid vector values, rather than simply classifying predefined angles and velocities. A comparison with other calorimetric sensors is presented in Table S1. By moving away from conventional bulky and complex flow sensors and introducing an asymmetric spiral thermal flow sensor, we demonstrated the feasibility of implementing a simple yet highly accurate sensor that fully integrates with embedded systems, enabling deep learning applications.

**Figure 5:**
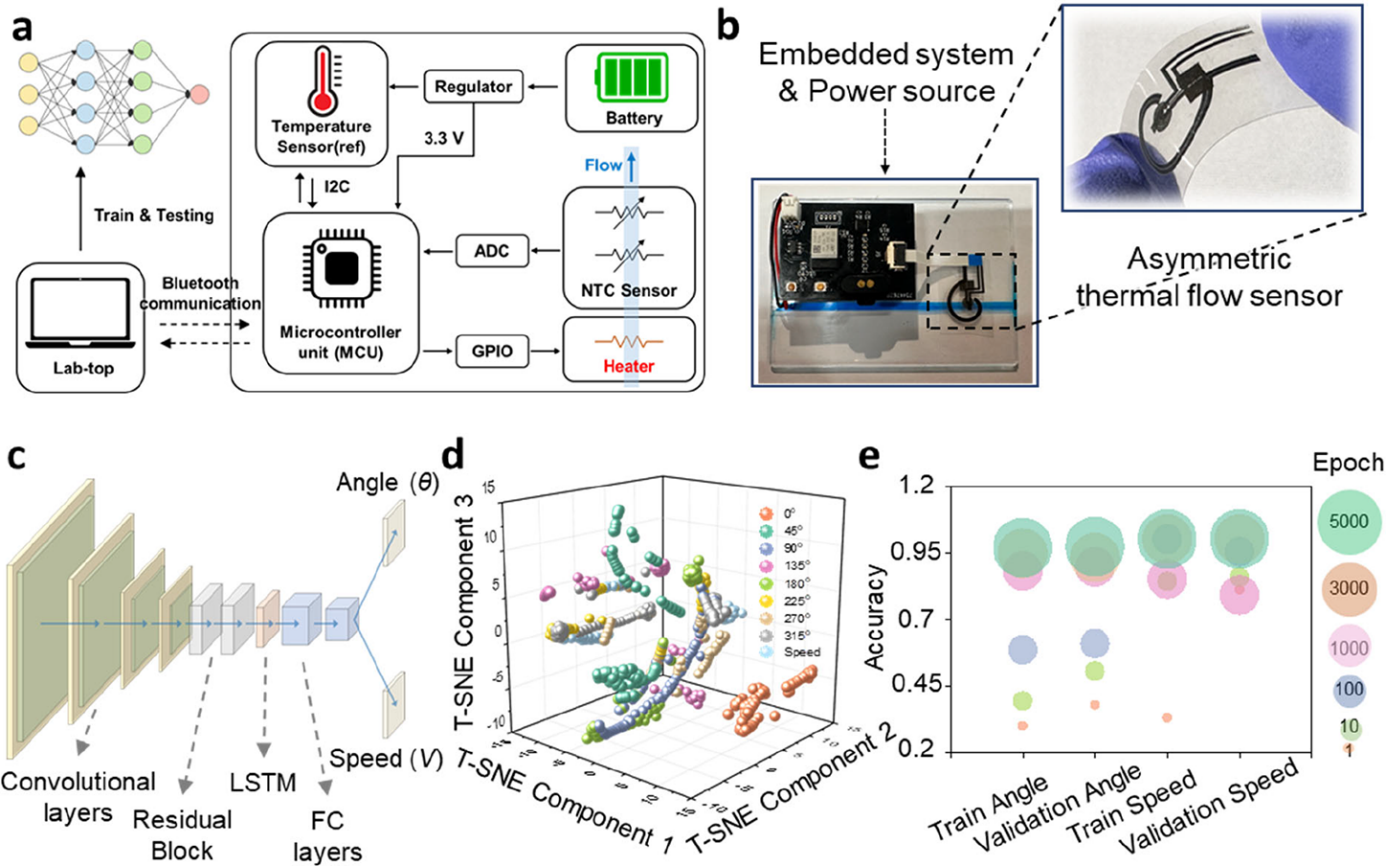
Schematic illustrating data processing via wireless transmission and deep learning. (a) Flowchart of the integrated flow sensor system, combining hardware and software for data acquisition and analysis. Resistance is measured using an ADC, with PWM-controlled heating and Bluetooth transmission for deep learning-based classification. (b) Photograph of the fabricated wireless module, including an embedded system, battery, and monolithic asymmetric thermal flow sensor. The inset shows a close-up of the sensor. (c) Schematic of the CNN-based deep learning framework for classifying flow speed and direction. The model incorporates CNNs, residual blocks, and LSTMs to capture spatial and temporal features, with fully connected layers for robust classification. (d) Scatter plot of resistance data visualized using t-SNE for flow speed and direction. High-dimensional data is mapped into 3D space, with points color-coded by angle class to highlight clustering patterns. (e) Deep learning analysis demonstrating classification accuracy of 100% for flow speed and 96% for flow direction. Training accuracy stabilized after approximately 5000 epochs.

## Conclusions

In this study, we developed a novel deep-learned monolithic asymmetric thermal flow sensor that integrates a laser-based selective sintering and reduction processes for fabricating heater, and temperature sensors in a thin-film configuration. This innovative design significantly minimizes flow distortion while ensuring precise flow speed and direction. Unlike conventional flow sensors that require complex arrays of multiple sensors, our system employs an asymmetric spiral temperature sensor surrounding the heater, enabling accurate flow direction detection through a single resistance change. In addition, a temperature sensor positioned directly beneath the heater effectively monitors the heater’s heat loss, providing a reliable indication of the flow velocity. The seamless integration of a wireless communication module enhances the real-time monitoring capability of the system, while the implementation of deep learning algorithms enables precise classification of flow speed and direction. Our experimental results demonstrate high performance: the deep learning model achieved 100% accuracy in classifying flow speed and 96% accuracy in determining flow direction from the sensor data. The laser-induced reduction sintering method proved effective in transforming NiO nanoparticles into functional Ni heater/electrode elements in situ, while preserving an NiO sensing region with stable NTC behavior. The sensor maintained consistent performance over repeated heating and cooling cycles, indicating excellent durability. By combining a highly sensitive monolithic asymmetric thermal flow sensor design with advanced data processing techniques, this system represents a significant advancement in flow sensing technology. Its compact, thin-film architecture is nonintrusive to the flow, making it highly adaptable to various industrial and environmental applications where precise fluid flow monitoring is critical. The deep learning-driven signal analysis further enhances its potential for intelligent automation and real-time process control. Moving forward, there are opportunities to improve and expand this work. Further miniaturization of the sensor could enable integration into even smaller or more complex fluid systems. Although water was used as the working fluid for initial validation in this study, the proposed sensor platform is expected to be applicable to other fluid types, including gases and high-viscosity liquids. This is because the sensing mechanism fundamentally depends on the thermal properties of the working fluid, such as thermal conductivity and specific heat capacity. Additionally, characterization based on a single temperature sensor positioned directly beneath the heater may inherently offer limited accuracy compared to multisensor calorimetric methods. However, this limitation is strategically addressed in our design through the integration of a sophisticated deep learning framework. The monolithic asymmetric spiral sensor layout simplifies the overall device architecture, minimizes flow interference, and reduces system complexity, thereby providing a suitable platform for deep learning integration. Furthermore, if the deep learning model is trained on a broader range of flow conditions and optimized to estimate continuous velocity and direction vectors instead of merely classifying discrete flow angles, the sensor system could evolve into a more scalable and versatile platform for flow analysis. This enhancement would significantly extend its applicability to complex fluidic environments and surpass the capabilities of conventional systems based on multiple independent temperature sensors. With these future developments, the monolithic asymmetric thermal flow sensor could find broad adoption in applications ranging from microfluidic diagnostics to large-scale industrial flow systems, providing intelligent, real-time flow vector monitoring with minimal impact on the flow itself.

## Materials and Methods

### Materials

All chemicals used in this study were of analytical grade and were used without further purification. For the synthesis of nickel oxide nanoparticle ink, nickel oxide (NiO, 99%, 10–20 nm) was purchased from US Research Nanomaterials, polyvinylpyrrolidone (PVP 10, MW 10,000) was purchased from Sigma-Aldrich, and ethanol (EtOH, 99.5%) was purchased from Samchun Chemicals.

### Synthesis of Nickel Oxide Nanoparticle Solution

Five g of PVP was dissolved in ethanol using a sonicator. After dissolution, 5 g of NiO nanoparticles were added, and the mixture was sonicated with a tip sonicator until the nanoparticles were fully dispersed. The dispersion was stirred magnetically at 70 °C for 24 h to dissolve completely. Following this, the mixture was centrifuged at 5000 rpm for 15 min. After centrifugation, the supernatant ethanol was removed. To the resulting precipitate, 5 mL of ethanol containing 0.8 g of PVP was added, and the mixture was sonicated until the nanoparticles were completely dispersed (Figure S16).

### NiO Nanoparticle Solution Coating

The slide glass (1 mm thick, Fisher) and the PET film (15 μm) were cleaned with ethanol. To prevent the PET film from lifting during the laser process, PDMS was spin-coated on the slide glass at 500 rpm for 30 s. The PET film was electrostatically attached to PDMS via corona treatment (High-Frequency Generator, Electro-Technic Products) and fixed along the edges with commercial tape (Scotch Magic Tape, 3M). A 400 μL of NiO nanoparticle solution was then dispensed onto the PET film and evenly coated using the doctor blading technique. After the NiO nanoparticle solution dried, the same coating process was repeated once more. Coated NiO nanoparticle solution was dried under the ambient conditions to obtain uniform NiO nanoparticle film. Since the ethanol-based ink is sensitive to ambient conditions such as humidity and temperature, which can influence the evaporation rate and resulting film uniformity, a high-viscosity formulation was employed by incorporating PVP. The increased viscosity helped suppress internal fluid flow and mitigate the coffee-ring effect, leading to the formation of uniform and crack-free films during drying.

### Patterning of the NiO Sensor and Heater

A 532 nm continuous-wave laser (Sprout-G 5W, Lighthouse Photonics) was used to reduce NiO for electrode formation and to remove PVP in the sensing channel. The laser system consists of an *f*-theta telecentric lens (*f* = 103 mm), a Galvano mirror (hurrySCAN II, Scanlab), and the aforementioned CW laser, which was utilized to induce reduction of nickel oxide nanoparticles and enhance adhesion with the substrate. The galvanometer scanner is integrated into the CAD software, enabling the control of laser power, scan speed, and tracing of paths to generate the desired pattern. In this study, the optimized laser power was 100 mW, and the scanning speed was 5 mm/s. The spacing between laser scanning lines was set to 20 μm for the electrode and 70 μm for the sensing channel. Although the hatch distance for the sensing channel was set to 70 μm, the actual width of the patterned region formed by laser irradiation was approximately 50 μm, due to thermal diffusion and material response during processing. The residual NiO film was removed by repeatedly immersing the substrate in deionized (DI) water. Following the cleaning process, the substrate was dried under ambient conditions.

### Temperature Sensitivity Measurements

The electrical resistance of the NiO temperature sensor was measured over a temperature range of 25–70 °C. The electrical resistance was measured by a sourcemeter (Keithley 2450). The temperature of the substrate was precisely controlled by a gas sensor measurement software (PMC4000).

### Characterization of the NiO Sensing Channel and Ni Electrode

UV–vis spectroscopy (V-770, JASCO) was used to measure the optical properties of the NiO sensing channel over the wavelength range of 200–750 nm. The measurement was performed in reflectance mode to determine the optical bandgap of the sensing material. Raman spectroscopy (Renishaw InVia Raman microscope) analysis was conducted to investigate changes in the crystalline characteristics resulting from the laser process. Atomic force microscopy (AFM, NX-10, Park Systems) was used to examine the surface morphology of the NiO sensing channel. SEM imaging and EDS analysis was conducted by field-emission scanning electronic microscopy (FE-SEM, Merlin Compact; Carl Zeiss NTS Ltd.) to investigate the shape and degree of reduction of the sensing channel and electrode.

### Flow Sensor Performance Measurements

The liquid flow rate was controlled by a syringe pump (NE-300, New Era Pump Systems, Inc.). The liquid flow was guided through a tube and a PDMS channel. The temperature of the substrate was controlled by a laser-patterned heater, which was powered by a power supply (Keithyley 2231A-30-3). To evaluate the performance of the heater, a power supply was used to apply controlled voltage and power at fixed intervals in both voltage and time, ensuring stable operation in terms of voltage and current. The heater was attached beneath a glass substrate, and an infrared camera was used to measure the temperature of the heater as a function of the applied voltage and time. The electrical resistance of the temperature sensor was measured and recorded in real time using an electrometer (Keithley 6514). To visualize the heat distribution and validate the thermal response of the device during flow, infrared thermal images were captured using an IR camera (FLIR A645 sc, Teledyne FLIR). Real-time data transmission via wireless communication was implemented using the MCU (STM32WB5MMG6TR). This MCU was used for Bluetooth transmission, collecting data at 1 s intervals. The heater was controlled using PWM. Its positive terminal was connected to a battery power supply to provide electrical power, while the negative terminal was connected to the drain of a MOSFET driven by the MCU, enabling power regulation. The PWM duty cycle was set to 81% to ensure stable heater operation. Simultaneously, resistance variations were monitored using the ADC functionality of the MCU. Communication with reference commercial temperature and humidity sensor was conducted via I2C. The data received via Bluetooth on a laptop was displayed in real time using a Python-based GUI.

## Supplementary Material









## Notes

Detailed information on the fabrication and optimization of the monolithic asymmetric thermal flow sensor, including laser-induced selective reduction processing of NiO ink; AFM imaging before and after laser processing showing the effect of laser intensity on nanoparticle sintering; variation in sheet resistance with laser power confirming the reduction of Ni, while optical band gap analysis further verifies the selective reduction of NiO; performance characterization including resistance changes under different heater power and flow rates, with theoretical and experimental comparisons; PCB circuit designed for wireless data transmission and experimental setup images; training loss curves for angle and speed classification, as well as accuracy variations over epochs, demonstrating the effectiveness of the sensor’s resistance-based learning approach (PDF)
Fabrication of an asymmetric thermal flow sensor using laser-induced selective reduction process (MP4)
Observation of heat distribution using an IR camera according to liquid flow speed (MP4)
Real-time wireless monitoring of resistance changes in a monolithic asymmetric thermal flow sensor (MP4)
