# Supplementary material for: A Deep-Learned Monolithic Nanoparticle Asymmetric Thermal Flow Sensor for Flow Vector Estimation
Source: ACS Nano. 2025 Aug 12;19(34):30961–72. doi: 10.1021/acsnano.5c07646 (PMC12410051; doi:10.1021/acsnano.5c07646)
Supplement: Supplementary file 1 [file nn5c07646_si_001.pdf]

# Supplementary materials

## A Deep-Learned Monolithic Nanoparticle Asymmetric Thermal Flow Sensor for Flow Vector Estimation

Huijae Park[1]†, Sangjin Yoon[1]†, Junhyuk Bang[1], Jiyong Ahn[1], Gyuho Choi[1], Dohyung Kim[1], JinKi Min[1], Jaeho Shin[2], Seung Hwan Ko[1,3,4,5*]

[1] Wearable Soft Electronics Lab, Department of Mechanical Engineering, Seoul National University, 1 Gwanak-ro, Gwanak-gu, Seoul, 08826, Korea
[2] Molecular Recognition Research Center, Korea Institute of Science and Technology (KIST), Seoul, 02792, South Korea
[3] Institute of Advanced Machinery and Design (SNU-IAMD), Seoul National University, Gwanak-ro, Gwanak-gu, Seoul 08826, Korea
[4] Institute of Engineering Research, Seoul National University, 1 Gwanak-ro, Gwanak-gu, Seoul 08826, Republic of Korea
[5] Interdisciplinary Program in Bioengineering, Seoul National University, Gwanak-ro, Gwanak-gu, Seoul, Korea

# Supporting Information

**The Supporting Information is available free of charge at ACS Publications website.**

Schematic illustration of the optical setup for NiO reduction; fabrication process of the asymmetric thermal flow sensor using laser-induced selective reduction; dual-side laser patterning of thermal flow sensor on PET substrates; AFM images of NiO before and after laser processing showing nanoparticle sintering; laser scan speed and power-dependent reduction morphology and post-cleaning substrate adhesion; sheet resistance variation of NiO films with laser power; resistance response of temperature sensors at varying distances from heater; CAD designs of spiral-shaped sensor, circular heater, and integrated configuration; microfluidic channel fabrication using PDMS and polyurethane sealing; temperature-resistance behavior at various hatch spacings indicating metallic and semiconducting characteristics; mechanical durability of sensors under bending stress; resistance change with heater power and flow rate including theoretical and experimental comparison; wireless data transmission circuit and experimental setup; deep learning training curves for angle and speed classification; validation accuracy trend and confusion matrix; preparation procedure of NiO nanoparticle dispersion using PVP-assisted sonication and centrifugation; summary and comparison of calorimetric sensor performance; real-time video demonstration of laser-induced sensor fabrication; infrared thermal distribution with flow rate; and wireless resistance monitoring in operating sensor.

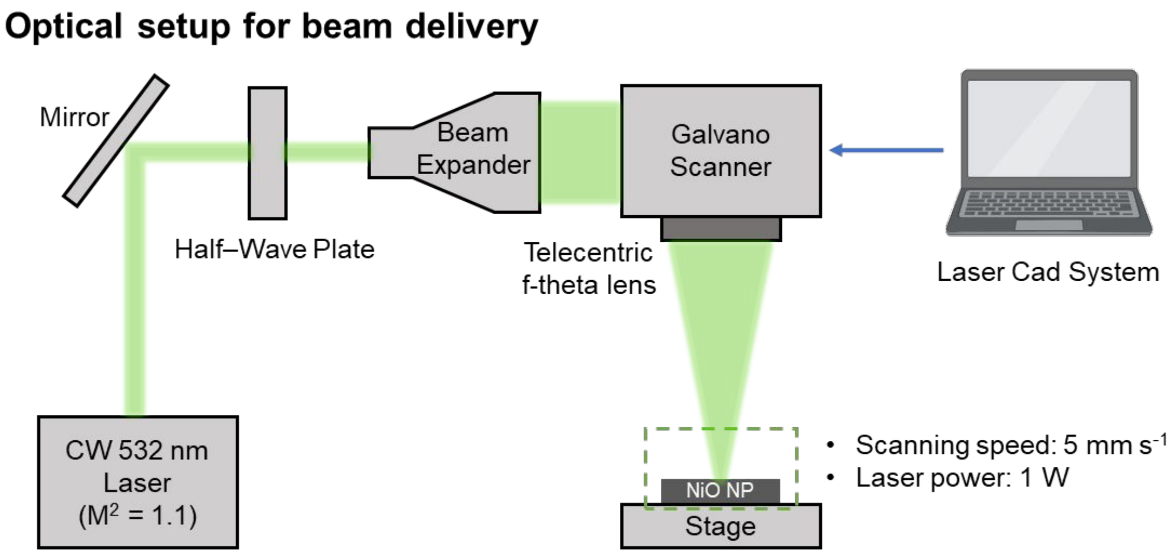


**Figure S1.** Schematic illustration of the optical setup used for NiO reduction processing. A CW 532 nm laser is directed through a beam expander and guided by a galvanometric scanner controlled by a computer. The focused laser beam is irradiated onto the sample mounted on a stage.

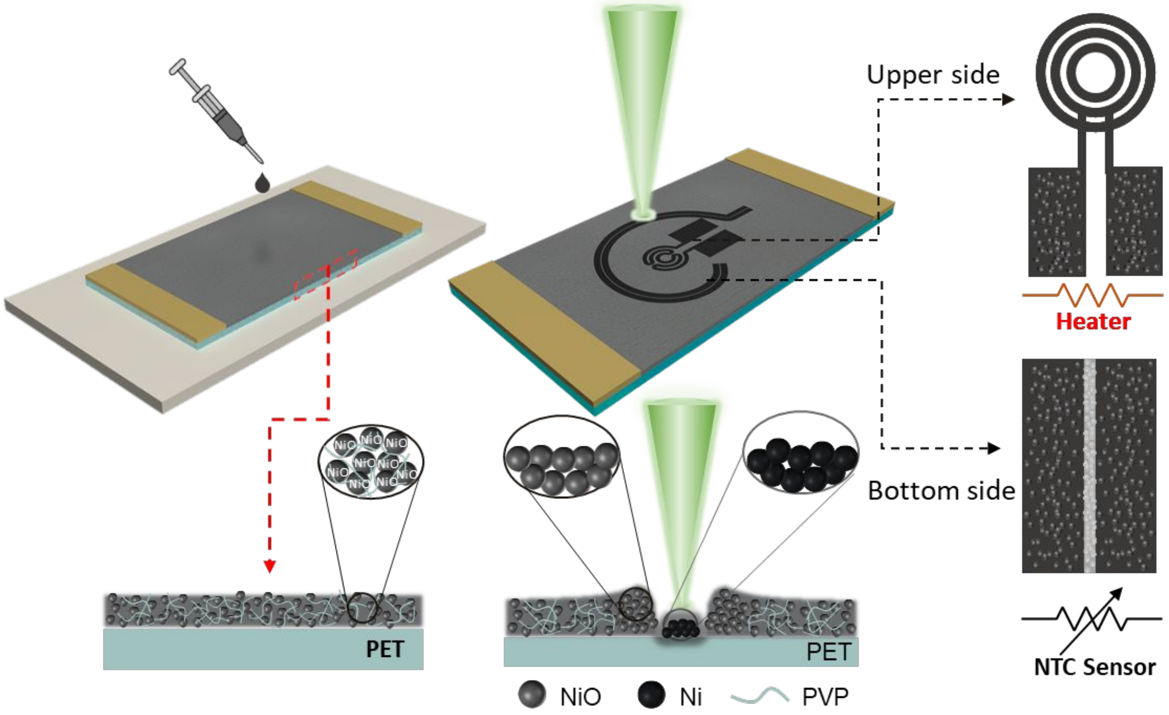


**Figure S2.** Fabrication of the asymmetric thermal flow sensor through laser-induced selective reduction processing of NiO nanoparticle ink. The heater, which applies heat to the top surface, and the temperature sensor, whose resistance varies with temperature, are patterned on the bottom surface.

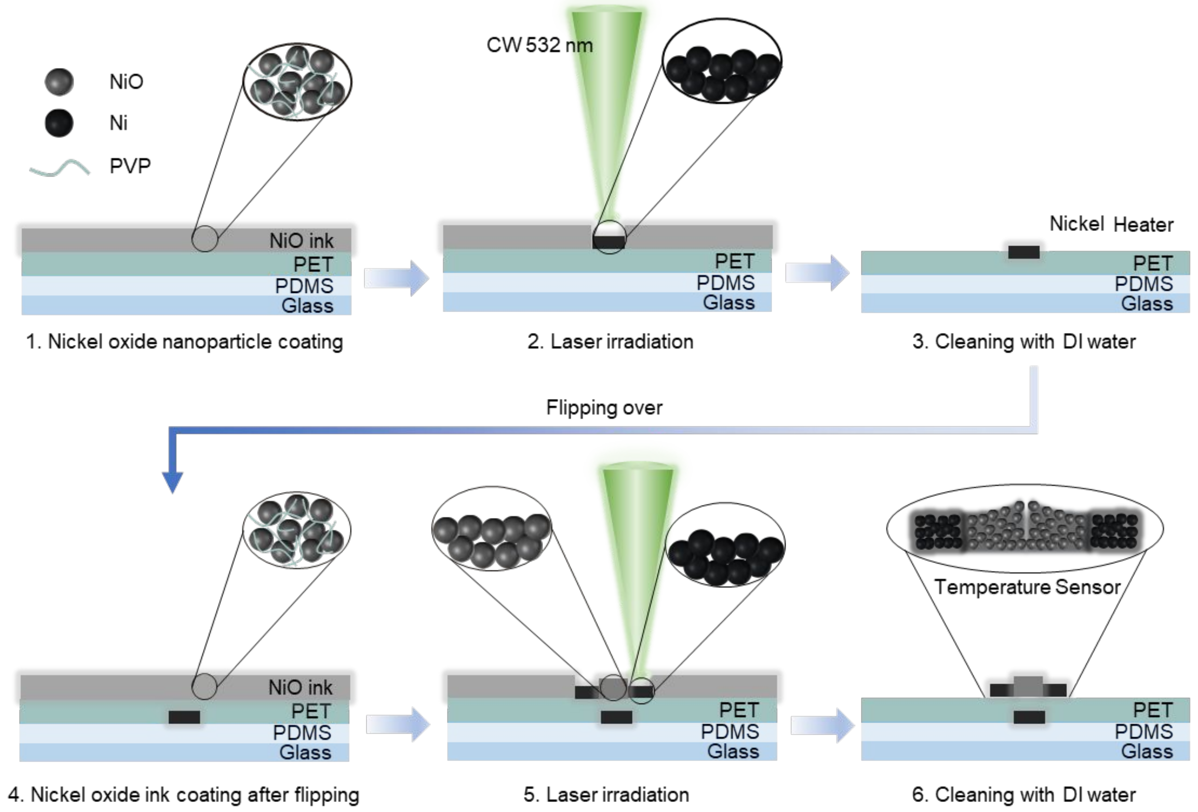


**Figure S3.** Schematic illustration of the laser reduction process for patterning NiO ink on both sides of a PET substrate. In the first step, NiO ink is coated on the top surface of the PET substrate and selectively reduced by a focused laser beam to form a conductive pattern. For double-sided patterning, the substrate is flipped, and the same process is applied to the opposite side to form an asymmetric temperature sensor.

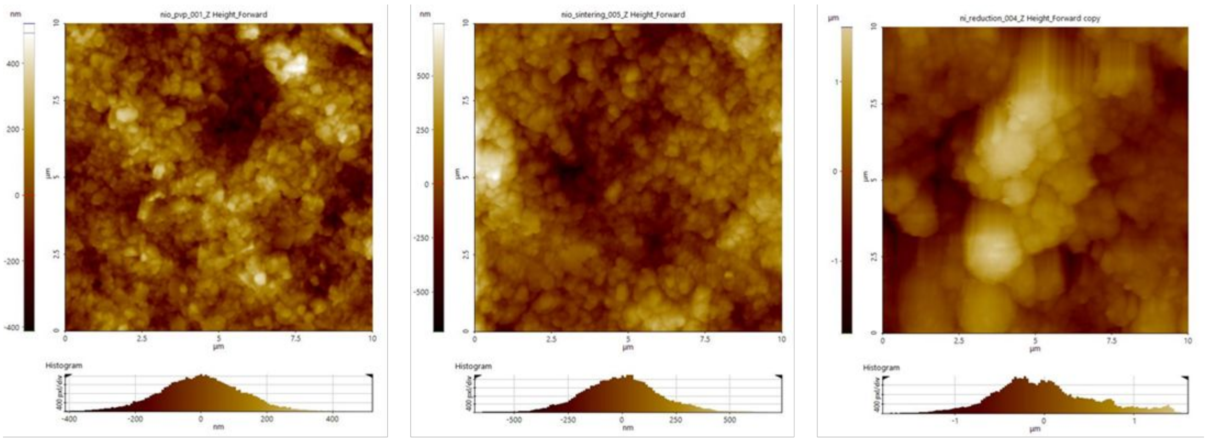

**Figure S4.** Atomic Force Microscopy (AFM) images of NiO before laser processing (left), sintered NiO (center), and reduced Ni (right) after laser processing. The root mean square roughness (Rq) values are 125.412 nm, 166.254 nm, and 568 nm, respectively, showing that as the laser intensity increases, the degree of nanoparticle sintering becomes more pronounced.

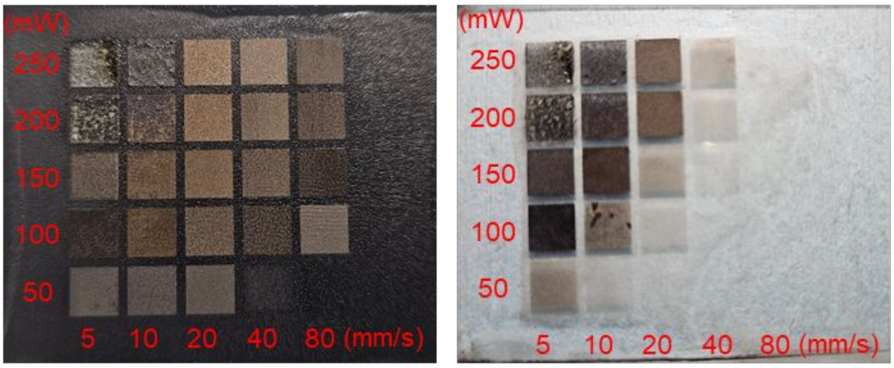


**Figure S5.** Laser reduction parameter study of NiO nanoparticles. (Left) Effect of laser scan speed and power on reduction morphology. (Right) Post-cleaning substrate indicating successful nanoparticle adhesion.

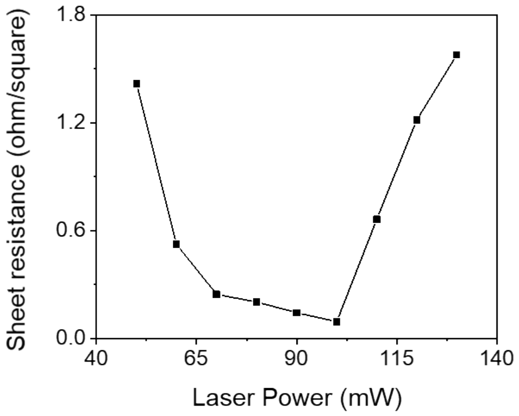


**Figure S6.** Variation in sheet resistance of the NiO film as a function of laser power. The laser speed was fixed at 5 mm/s, and at a power of 100 mW, the reduction of Ni was observed.

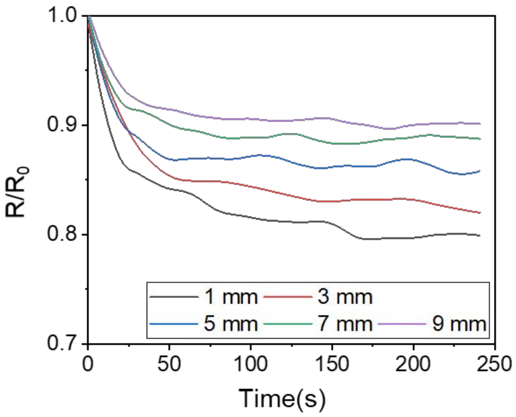


**Figure S7.** Normalized resistance changes of the temperature sensor as a function of time at varying distances from the heater. The results indicate that sensors positioned closer to the heater exhibit a larger change in resistance, confirming a stronger thermal response with decreasing distance.

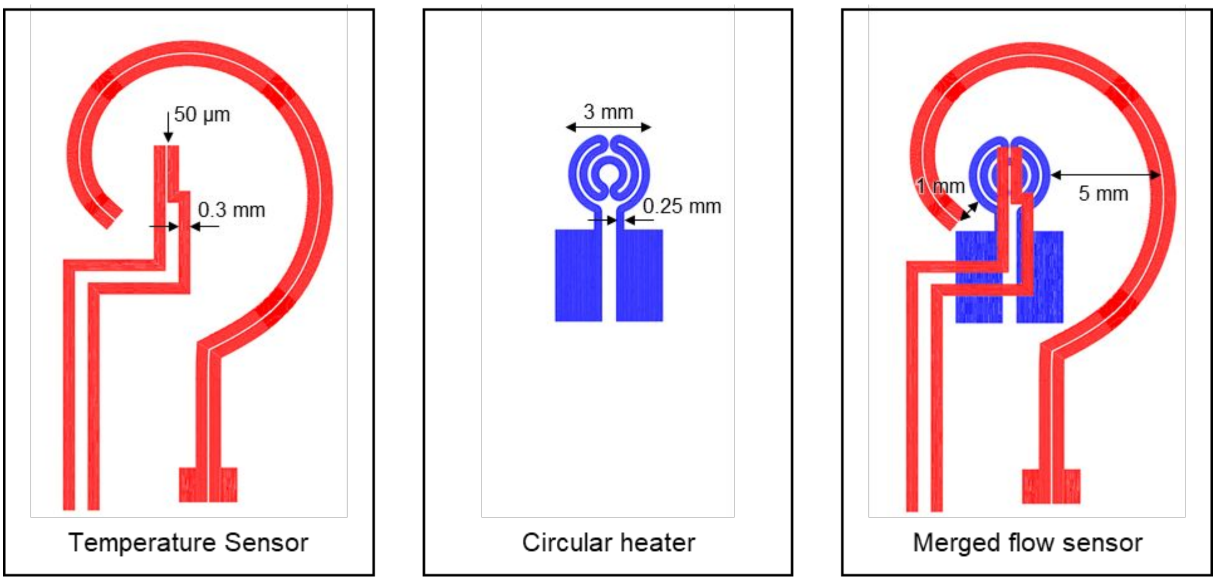


**Figure S8.** CAD designs of the laser-fabricated heater and temperature sensor. (Left) Spiral-shaped temperature sensor pattern. (Center) Circular heater. (Right) Overlaid configuration showing the integrated heater and sensor.

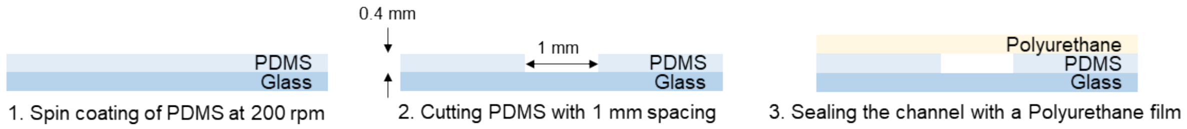


**Figure S9.** Schematic illustration of the microfluidic channel fabrication process. (Left) A PDMS layer bonded to a glass substrate. (Center) A channel cavity formed within the PDMS. (Right) A polyurethane film sealed on top of the PDMS to enclose the channel, completing the microfluidic channel.

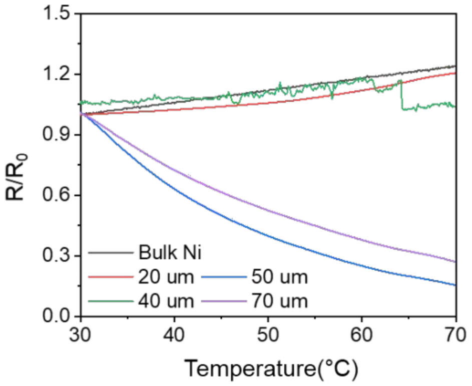


**Figure S10.** Temperature-dependent resistance change of the temperature sensor measured at various hatch spacings. When the hatch spacing is below 50 µm, the sensor exhibits metallic behavior, with resistance increasing as temperature rises. In contrast, hatch spacings above 50 µm result in a negative temperature coefficient response.

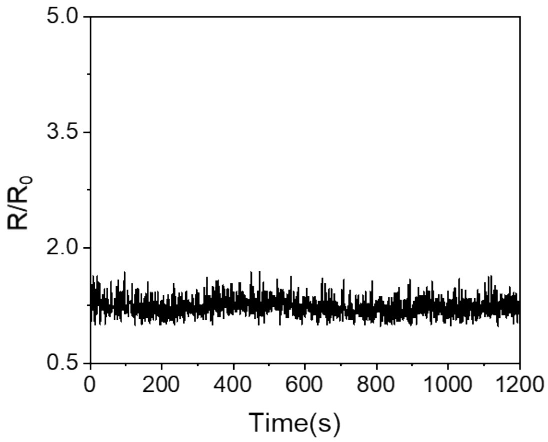


**Figure S11.** Resistance change of a temperature sensor attached to a PET substrate under mechanical bending. The device was subjected to 300 bending cycles with a radius of 5.18 mm.

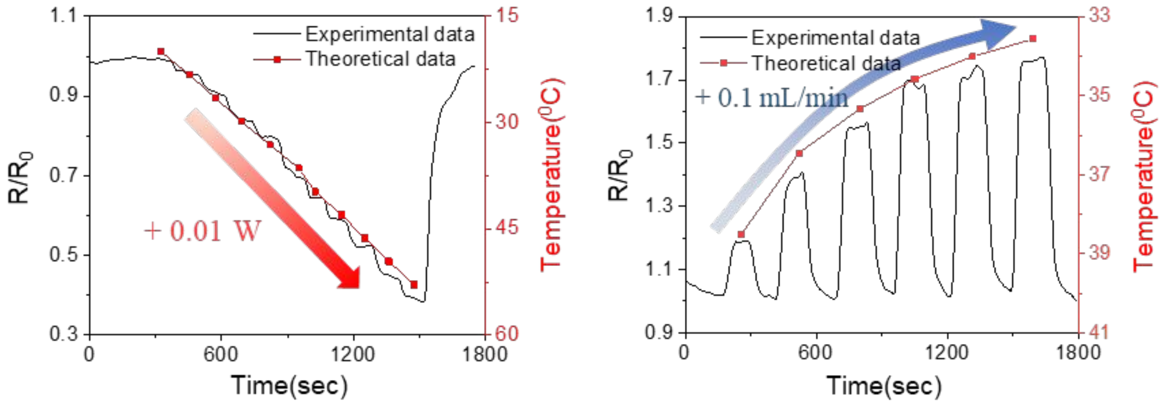


**Figure S12.** The left figure shows resistance changes with varying heater power while keeping the flow rate constant at 0.1 mL/min, and the right figure shows resistance changes with varying flow rate while keeping heater power constant at 0.13 W. The red line represents theoretical values, and the black line shows experimental data.

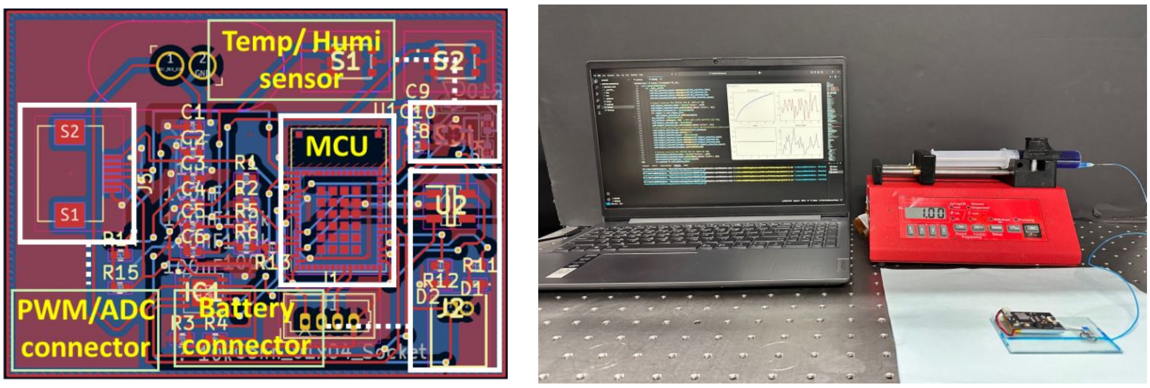


**Figure S13.** PCB circuit design and experimental setup photos for wirelessly transmitting data from an asymmetric thermal flow sensor.

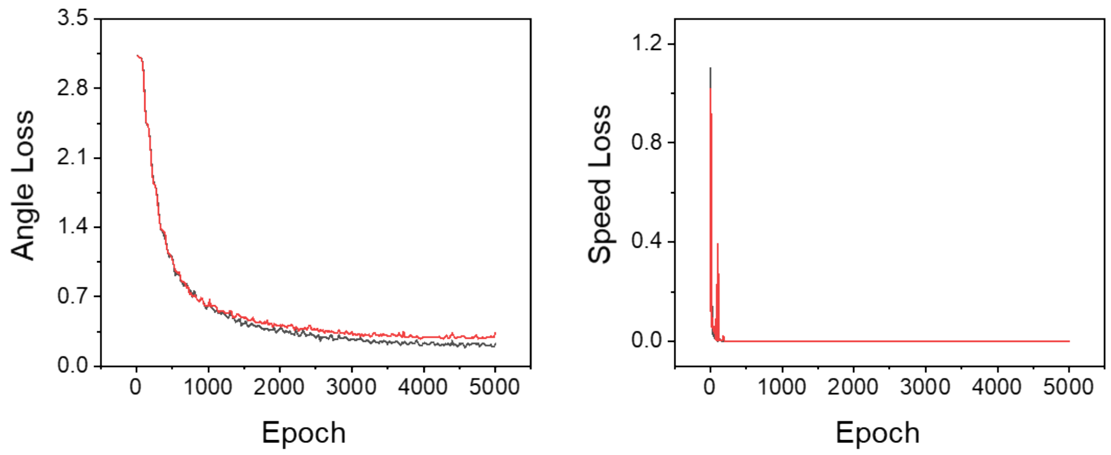


**Figure S14**. Training loss curves for angle (left) and speed (right) for the thermal flow sensor. The angle was classified by learning the resistance changes of the asymmetric sensor, while the speed was classified by learning the resistance changes of the symmetric sensor under the heater.

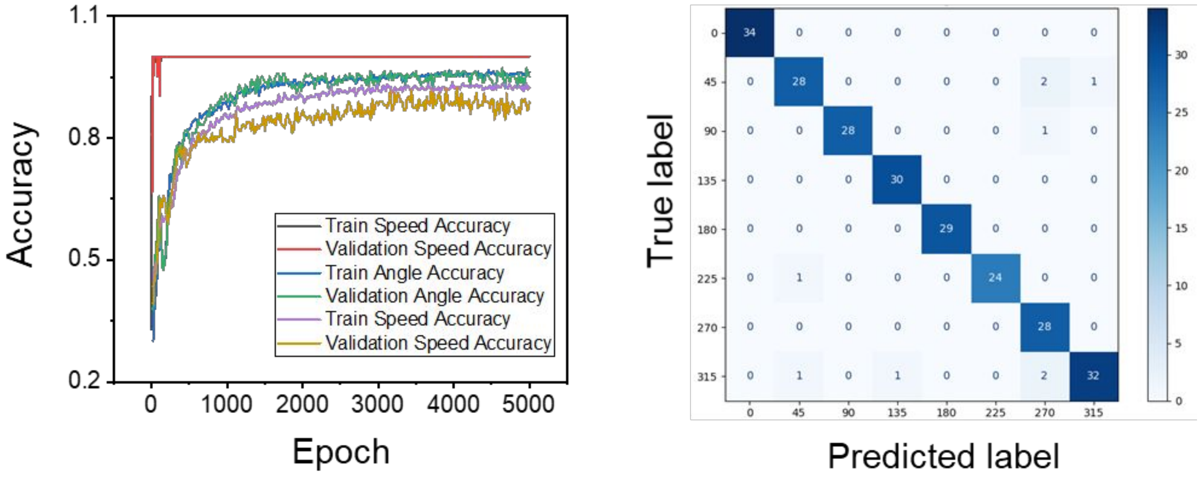


**Figure S15.** The graph on the left shows the changes in accuracy over epochs during the training process using the resistance data from the asymmetric thermal flow sensor. The graph on the right is a confusion matrix that quantitatively illustrates the validation accuracy.

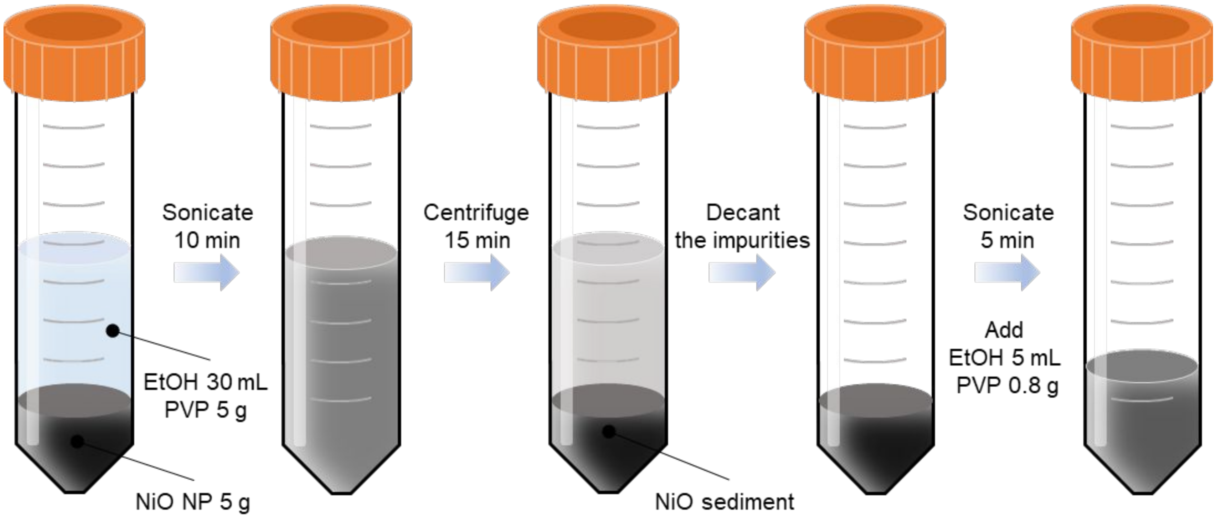


**Figure S16.** Preparation process of NiO nanoparticle dispersion. PVP was dissolved in ethanol and mixed with NiO nanoparticles, followed by tip sonication. The mixture was centrifuged, and the supernatant was removed. The precipitate was redispersed in ethanol containing PVP and sonicated again.

**Table S1. Summary and comparison of calorimetric sensors.**

| Category | This work | Xu et al., 2023[1] | Xu et al., 2019[2] | Gong et al., 2024[3] | Barmpakos et al., 2020[4] | Barmpakos et al., 2019[5] |
|---|---|---|---|---|---|---|
| No. of heater | 1 heater | 4 heaters | 1 heater | 1 heater | 1 heater | 1 heater |
| No. of temperature sensor | 1 temperature sensor (flow speed)<br>1 temperature sensor (flow direction) | 8 temperature sensors | 8 temperature sensors | 12 temperature sensors | 4 temperature sensors | 4 temperature sensors |
| Sensing material | Nickel oxide | Laser-induced graphene (LIG) | $SnO_2$ nanoparticle / single-walled carbon nanotubes (SWCNT) | $VO_x$ | $BaTiO_3$ + Activated carbon + Thermoset polymer system (PTC) | Pt |
| Electrode material<br>Fabrication method | Laser-reduced Ni<br>Laser-induced selective reduction | Ag<br><br>$CO_2$ laser direct writing | Ag<br><br>Electrode: screen printing + curing<br>Sensor: drop-casting + baking | Cr, Au<br><br>MEMS fabrication | Ag ink<br><br>Screen printing | Cu<br><br>Photolithography |
| Measurable flow speed | 0-12.5 mm/s | 0 – 25 m/s | 0 – 3 m/s | 0.11 mm/s ~ 30 m/s | 0 – 25 SLPM | 0 – 25 SLPM |

## Supporting Note 1

The asymmetric thermal flow sensor has the potential to be used in various applications, as it can measure the direction and velocity of fluid flow and is well-suited for small channels such as microchannels due to its compact size. Additionally, its ability to measure flow externally without being immersed in the fluid helps minimize contamination and interference. Possible applications include biomedical and lab-on-a-chip devices for microfluidic flow monitoring and drug delivery systems. In industrial and process control, it could be utilized for coolant flow monitoring, semiconductor fabrication, and leak detection in microfluidic pipelines. Furthermore, it may have applications in wearable and IoT devices for sweat analysis and smart fluid management in industrial and home automation systems.

## Supporting Note 2

When attaching PET to a glass substrate after plasma treatment and coating it with NiO ink for heater laser patterning, a lifting phenomenon occurs in the PET. This issue causes misalignment of the focal point when patterning the sensor on the backside, making precise sensor patterning difficult. To address this problem, a thin layer of PDMS was coated onto the glass substrate before plasma-treating and attaching the PET, followed by laser patterning. Since the PDMS layer securely adheres to the PET, the lifting issue is prevented, ensuring that the focal point remains aligned during backside laser patterning, allowing for precise patterning. Additionally, alignment markers were placed on the laser stage, enabling accurate substrate positioning during the process.

## Movie S1. Fabrication of an asymmetric thermal flow sensor using laser-induced selective phase transformation.

PDMS was spin-coated on a glass substrate at 500 rpm and then cured at a high temperature. A PET layer was attached on top, and a 100 μm thick spacer was created using tape. NiO ink was then applied

using bar coating, followed by laser patterning. The laser patterning was performed at a speed of 5 mm/s, a power of 100 mW, and a defocused focal length of 0.5 mm. The areas directly exposed to the laser received sufficient heat for reduction, converting NiO into Ni. In the surrounding areas indirectly exposed to the laser, the PVP melted, causing the NiO to sinter and function as a temperature sensor. The top surface of the PET was patterned as a heater, while the bottom surface was patterned as a sensor.

**Movie S2. Observation of heat distribution using an IR camera according to liquid flow rate.**

PDMS was spin-coated on a glass substrate at 150 rpm and then cured at a high temperature. A flow channel with a 1 mm gap was created, and a thin polymer film was attached on top. A laser-patterned heater was then placed on this surface, and heat was applied by supplying voltage. Using a syringe pump, water was flowed at rates of 0, 0.1, 0.2, and 0.3 ml/min, and the heat distribution under each condition was measured using an IR camera.

**Movie S3. Real-time monitoring of resistance changes in an asymmetric thermal flow sensor via wireless communication.**

A wireless communication embedded system circuit was designed to measure the resistance variation of an asymmetric thermal flow sensor. The heater was controlled using pulse-width modulation (PWM), and the sensor's resistance changes were measured using an analog-to-digital converter (ADC). The resistance variation data was transmitted via Bluetooth, allowing for the acquisition of liquid flow velocity and direction data. The wirelessly transmitted data was monitored in real-time through a graphical user interface (GUI) and stored. The collected data was then utilized for deep learning-based classification of flow velocity and direction.

## Reference


1 Xu, K. *et al.* Laser direct writing of flexible thermal flow sensors. *Nano Letters* **23**, 10317-10325 (2023).

2 Xu, K. *et al.* Highly precise multifunctional thermal management-based flexible sensing sheets. *ACS nano* **13**, 14348-14356 (2019).

3 Gong, Z. *et al.* Flexible calorimetric flow sensor with unprecedented sensitivity and directional resolution for multiple flight parameter detection. *Nature Communications* **15**, 3091 (2024).

4 Barmpakos, D. *et al.* A fully printed flexible multidirectional thermal flow sensor. *Flexible and Printed Electronics* **5**, 035005 (2020).

5 Barmpakos, D., Famelis, I. T., Moschos, A., Marinatos, D. & Kaltsas, G. Design and evaluation of a multidirectional thermal flow sensor on flexible substrate. *Journal of Sensors* **2019**, 8476489 (2019).
